# Binding Pattern and Structural Interactome of the Anticancer Drug 5-Fluorouracil: A Critical Review

**DOI:** 10.3390/ijms25063404

**Published:** 2024-03-17

**Authors:** En-Shyh Lin, Cheng-Yang Huang

**Affiliations:** 1 Department of Beauty Science, National Taichung University of Science and Technology, Taichung City 403, Taiwan; 2 Department of Biomedical Sciences, Chung Shan Medical University, Taichung City 402, Taiwan; 3 Department of Medical Research, Chung Shan Medical University Hospital, Taichung City 402, Taiwan

**Keywords:** 5-fluorouracil, interactome, anticancer, binding mode, crystal structure, SSB, dihydroorotase, dihydropyrimidinase, PISA, PLIP

## Abstract

5-Fluorouracil (5-FU) stands as one of the most widely prescribed chemotherapeutics. Despite over 60 years of study, a systematic synopsis of how 5-FU binds to proteins has been lacking. Investigating the specific binding patterns of 5-FU to proteins is essential for identifying additional interacting proteins and comprehending their medical implications. In this review, an analysis of the 5-FU binding environment was conducted based on available complex structures. From the earliest complex structure in 2001 to the present, two groups of residues emerged upon 5-FU binding, classified as P- and R-type residues. These high-frequency interactive residues with 5-FU include positively charged residues Arg and Lys (P type) and ring residues Phe, Tyr, Trp, and His (R type). Due to their high occurrence, 5-FU binding modes were simplistically classified into three types, based on interactive residues (within <4 Å) with 5-FU: Type 1 (P-R type), Type 2 (P type), and Type 3 (R type). In summary, among 14 selected complex structures, 8 conform to Type 1, 2 conform to Type 2, and 4 conform to Type 3. Residues with high interaction frequencies involving the N1, N3, O4, and F5 atoms of 5-FU were also examined. Collectively, these interaction analyses offer a structural perspective on the specific binding patterns of 5-FU within protein pockets and contribute to the construction of a structural interactome delineating the associations of the anticancer drug 5-FU.

## 1. Introduction

Pyrimidine nucleotides are essential for a vast number of biological processes such as the synthesis of RNA, DNA, phospholipids, and glycogen and the sialylation and glycosylation of proteins [1]. The accurate synthesis of nucleotides is a critical process for the survival and proliferation of both eukaryotic and prokaryotic cells [2]. Structural alterations to nucleobases have the potential to exert substantial impacts, leading to potent biological effects. Derivatives of uracil, particularly in the realm of anticancer [3], antiviral [4], antibacterial [5], anti-inflammatory [6], and antitumor activities [7,8], have found longstanding utility. One notable example in this category is the FDA-approved anticancer agent, 5-fluorouracil (5-FU) [9]. In 5-FU, the hydrogen at the C5 position of uracil is replaced by a fluorine atom, resulting in a fluoropyrimidine configuration. This modification empowers 5-FU to effectively target the enzyme thymidylate synthase (TSase) for anticancer chemotherapy [10]. Despite the continuous emergence of novel agents in the field of drug development, 5-FU remains a cornerstone in the arsenal of chemotherapeutic modalities, playing a prominent role in systemic treatments for various cancers such as those affecting the gastrointestinal tract, breast, head, and neck [10].

The cytotoxic effects of 5-FU arise from its ability to impede the operation of TSase, induce RNA miscoding, and activate apoptosis [10]. Upon entering cells, 5-FU undergoes catalysis by several enzymes to produce 5-fluorouridine monophosphate, 5-fluorodeoxyuridine triphosphate, and 5-fluorouridine triphosphate. These pyrimidine antimetabolites inhibit TSase and/or mis-incorporate into RNA and DNA, leading to cell death and providing a basis for anticancer chemotherapy.

In the catabolic pathway, 5-FU is converted into dihydrofluorouracil by dihydropyrimidine dehydrogenase, with most of it being degraded in the liver [11,12]. This leads to the formation of α-fluoro-β-alanine and α-fluoro-β-ureido propionic acid, which are excreted through the kidneys. However, the oral administration of 5-FU exhibits poor bioavailability due to variability in dihydropyrimidine dehydrogenase activity [12]. To address severe toxicities in patients with dihydropyrimidine dehydrogenase deficiency, several 5-FU prodrugs have been developed. Toxicities associated with 5-FU have also been reported in asymptomatic patients with dihydropyrimidinase deficiency undergoing anticancer therapy [13]. These patients suffered from severe toxicity, including death, after treatment with 5-FU [13]. Additionally, the microbiota can influence the host response to 5-FU [14]. For instance, active gut microbiota capable of producing bromovinyluracil can increase systemic concentrations of 5-FU, leading to adverse effects [14]. Therefore, a comprehensive interactome of 5-FU is essential for detailed clinical pharmacokinetic and toxicity analyses. Currently, more than a dozen proteins, some characterized as probable antituberculosis targets of 5-FU [15,16], can bind and respond to 5-FU. The identification of new 5-FU-interacting proteins and understanding their binding modes for repurposing the clinical use of 5-FU warrant further research.

Introduced as an anticancer agent in the late 1950s, 5-FU remains one of the most widely prescribed chemotherapeutics, with an estimated 2 million people worldwide receiving 5-FU or one of its prodrug forms (e.g., capecitabine) each year [17]. Hence, exploring how 5-FU specifically binds to proteins is crucial for discovering additional 5-FU interacting proteins and understanding their medical potentials. This review consolidates and discusses the current knowledge on the binding modes of 5-FU to proteins based on available structural information, encompassing 23 structures of 5-FU-complexed proteins, of which 14 structures are unique and nonredundant.

## 2. Crystal Structures of 5-FU Complexes in the Protein Data Bank (PDB)

There are 23 structures of proteins complexed with 5-FU available in the Protein Data Bank (PDB) (Table 1). These structures can be broadly categorized into four groups based on their biological functions: (1) Reductase: dihydropyrimidine dehydrogenase; (2) Transferase: uridine phosphorylase, uracil phosphoribosyltransferase, RNA-dependent RNA polymerase, pyrimidine operon regulatory protein PyrR, and catalytic subunit alpha of phosphatidylinositol 4,5-bisphosphate 3-kinase; (3) Hydrolase: rRNA N-glycosidase, uracil-DNA glycosylase, dihydropyrimidinase, dihydroorotase, and hydroxydechloroatrazine ethylaminohydrolase; and (4) DNA binding protein: SsbA and SsbB. To avoid redundancy, we specifically reviewed the following structures that are complexed with 5-FU: PDB IDs 1H7X, 1UPF, 3NAI, 3NBQ, 4O0O, 4WRY, 5IAO, 5SXC, 6KLK, 6L0B, 7DEP, 7YM1, 8GVZ, and 8IS4. For consistency and a systematic analysis, interactions, including hydrogen bonding between the protein and 5-FU in these structures, are re-evaluated using the PISA (Protein Interfaces, Surfaces, and Assemblies; https://www.ebi.ac.uk/pdbe/pisa/, accessed on 4 February 2024) [18] and PLIP (the protein–ligand interaction profiler; https://plip-tool.biotec.tu-dresden.de, accessed on 4 February 2024) [19] software. Interactions within a contact distance (<4 Å) between the protein and 5-FU in each binding mode are also elucidated. The sizes of the 5-FU binding pockets were systematically analyzed using CASTp 3.0 [20] or manually measured to compare the dimensions of the binding cavities (Table 2). Based on the re-evaluated results, the patterns were classified into three types of the 5-FU binding modes (Table 3).

## 3. The Binding Mode of 5-FU

### 3.1. Dihydropyrimidine Dehydrogenase

**Figure 1:**
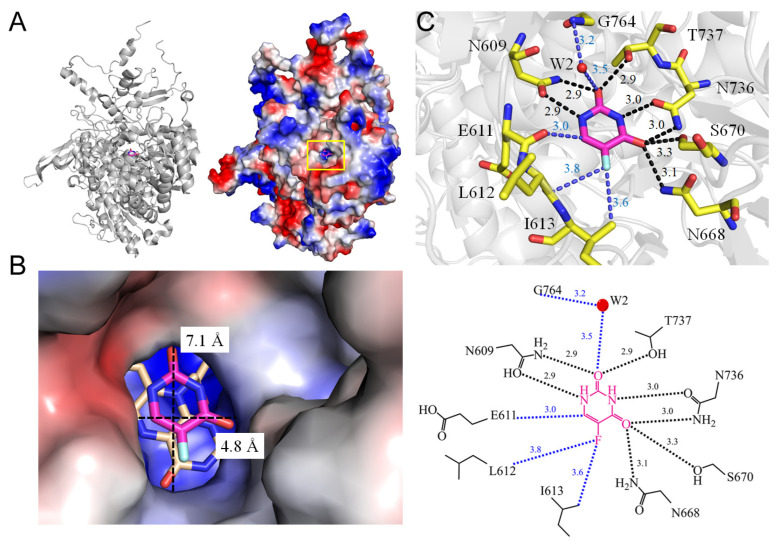
Crystal structure of dihydropyrimidine dehydrogenase complexed with 5-FU. (**A**) The dihydropyrimidine dehydrogenase complex (PDB ID 1H7X). 5-FU is colored in light magenta (boxed in yellow). The surface charge distribution pattern is also shown. Electrostatic surface potentials are colored red and blue for negative and positive charges, respectively. The 5-FU binding site is highlighted in gold. (**B**) The binding cavity. The size of the 5-FU binding pocket was manually measured (the dashed line) to compare the dimensions (x, y) of the binding cavities. In this structure, 5-FU is bound adjacent to the cofactor FMN, and the π–π interaction with FMN takes place for 5-FU. (**C**) The 5-FU binding mode. The binding site of 5-FU within dihydropyrimidine dehydrogenase is unveiled through the complex structure of 5-FU-bound dihydropyrimidine dehydrogenase. Residues engaging with 5-FU within the contact distance (<4 Å) are colored in yellow. The interactive distances are indicated (Å). For clarity, a depiction of the binding mode is also shown, with hydrogen bonding highlighted in black.

Dihydropyrimidine dehydrogenase catalyzes the initial step, which is the NADPH-dependent reduction in uracil and thymine to the corresponding 5,6-dihydropyrimidines, in pyrimidine catabolism [21]. This dimeric flavoprotein with four iron–sulfur clusters has become a controlled inhibition target for cancer therapy and treating solid tumors [22]. The enzyme represents the rate-limiting step responsible for the rapid breakdown of the chemotherapeutic drug 5-FU. Deficiency in dihydropyrimidine dehydrogenase activity can lead to severe and potentially fatal toxicity, with nearly 30% of patients with reduced activity experiencing severe toxicity during chemotherapy [9,11,23]. The co-crystal structure of dihydropyrimidine dehydrogenase from pig liver (*Sus scrofa*) has been resolved at a 1.9 Å resolution (PDB ID 1H7X) [24]. In this structure, 5-FU is bound adjacent to the cofactor FMN, and the π–π interaction with FMN takes place for 5-FU. The interaction involves hydrogen bonds with three asparagine side chains (N609, N668, and N736) and the side chains of S670 and T737 (Figure 1). G764 also interacts with 5-FU through water-molecule-mediated hydrogen bonding. Although the fluorine substituent of the drug does not directly contact the enzyme, it may interact with L162 and I163 at contact distances of 3.8 Å and 3.6 Å, respectively. The existence of this binding pocket explains the enzyme’s ability to tolerate other, bulkier substituents at the 5-position of the pyrimidine ring. This complex structure not only provides insights into the architecture of the drug-recognition site, but also allows predictions of risk variants of dihydropyrimidine dehydrogenase for drug toxicity. Furthermore, it offers valuable information about the structural basis of enzyme deficiencies observed in cancer patients due to point mutations in the human dihydropyrimidine dehydrogenase gene. Additionally, it serves as a framework for the structure-assisted design of new anti-cancer drugs.

### 3.2. Uracil Phosphoribosyltransferase

**Figure 2:**
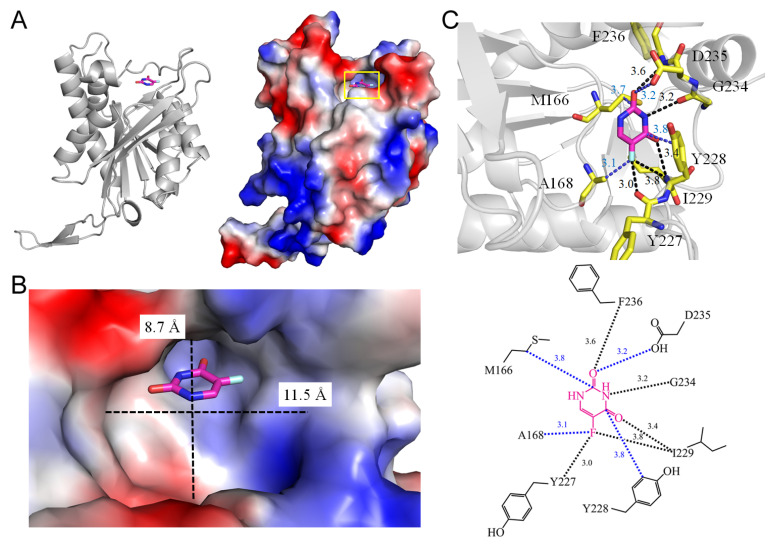
Crystal structure of uracil phosphoribosyltransferase complexed with 5-FU. (**A**) The uracil phosphoribosyltransferase (PDB ID 1UPF). (**B**) The binding cavity. (**C**) The 5-FU binding mode. Residues engaging with 5-FU within the contact distance (<4 Å) are colored in yellow. The interactive distances are indicated (Å). For clarity, a depiction of the binding mode is also shown, with hydrogen bonding highlighted in black.

Uracil phosphoribosyltransferase catalyzes the transfer of a ribosyl phosphate group from alpha-D-5-phosphoribosyl-1-pyrophosphate to the N1 nitrogen of uracil [25]. In the opportunistic pathogen *Toxoplasma gondii*, uracil phosphoribosyltransferase presents itself as a rational target for antiparasitic drug designs [26]. The absence of uracil phosphoribosyltransferase activity in humans, coupled with the observation that *T. gondii* uracil phosphoribosyltransferase binds various pyrimidine analogs, including 5-FU, known for its documented antitoxoplasmal activity, underscores the potential of *T. gondii* uracil phosphoribosyltransferase as a robust target for structure-based prodrugs and drug designs. The enzyme converts 5-FU, a prodrug, to the nucleotide level where it becomes toxic to the parasite, acting as a subversive substrate [26]. Uracil phosphoribosyltransferase increases the conversion of 5-FU into its active metabolites, thereby enhancing its cytotoxic effects for anticancer therapy. Regrettably, 5-FU also exhibits high toxicity in certain human populations, limiting its applicability. To facilitate structure-based prodrug design studies against toxoplasmosis, the crystal structure of *T. gondii* uracil phosphoribosyltransferase bound to the prodrug 5-FU has been elucidated (PDB ID 1UPF) [27]. The interaction of 5-FU includes hydrogen bonds with the main chains of Y227, I229, G234, and F236 (Figure 2). Y227 and I229, in collaboration with A168 (at a contact distance of 3.1 Å), interact with the fluorine substituent of 5-FU, determining the binding specificity. Given the substantial sequence homology among all uracil phosphoribosyltransferases, the *T. gondii* uracil phosphoribosyltransferase structure stands as a potential model for other uracil phosphoribosyltransferases.

### 3.3. RNA-Dependent RNA Polymerase

**Figure 3:**
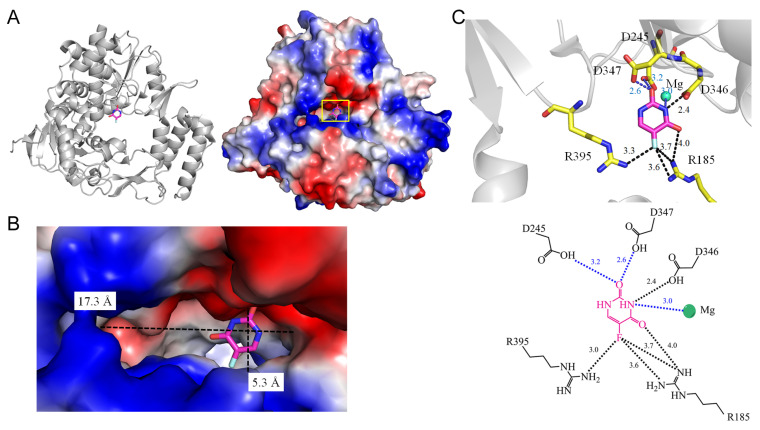
Crystal structure of RNA-dependent RNA polymerase complexed with 5-FU. (**A**) The RNA-dependent RNA polymerase complex (PDB ID 3NAI). (**B**) The binding cavity. (**C**) The 5-FU binding mode.

RNA-dependent RNA polymerases play a crucial role in catalyzing the formation of phosphodiester bonds between ribonucleotides in an RNA template-dependent manner [28]. In infected cells, these enzymes typically associate with other virus-encoded and host-encoded proteins that modulate RNA polymerization activity and template specificity. There is an abundance of three-dimensional structural information available for RNA-dependent RNA polymerases from various families of positive-stranded and double-stranded RNA viruses [29,30]. The co-crystal structure of the murine norovirus-1 RNA-dependent RNA polymerase bound to 5-FU has been elucidated (PDB ID 3NAI) [31]. Noroviruses, belonging to the *Caliciviridae* family of single-stranded positive-sense RNA viruses [32], are responsible for widespread outbreaks of acute gastroenteritis. They represent one of the most common causes of foodborne illness and a major public health concern, with no available vaccine or antiviral treatment options. The RNA-dependent RNA polymerase of noroviruses is a pivotal enzyme responsible for the transcription and replication of the viral genome. The complexed structure with 5-FU provides insights into the molecular basis of low fidelity and inhibitory activities on viral replication [31]. An essential metal ion in this enzyme interacts with D245 and D346. 5-FU interacts with R185, D346, D347, and R395 (Figure 3). The fluorine moiety of 5-FU forms hydrogen bonds with R185 (3.6 Å) and R395 (3.4 Å), which are highly conserved in RNA-dependent RNA polymerases. The metal ion at the active site is tetrahedrally coordinated to the carboxyl groups of D245 and D346, the hydroxyl group of 5-FU, and a water molecule. Therefore, D245, D346, and D347 form a network of interactions within the active site through a metal ion and a water molecule, mediating interactions between 5-FU and active-site residues to stabilize the ligand-bound structure. The structural insights gained from this RNA-dependent RNA polymerase complexed with 5-FU contribute to a better understanding of norovirus replication and aid in the design of novel therapeutic agents against this significant pathogen.

### 3.4. Uridine Phosphorylase

**Figure 4:**
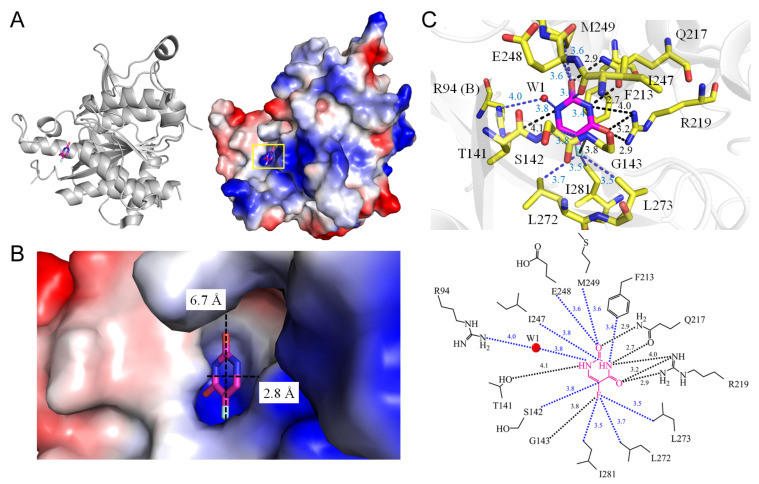
Crystal structure of uridine phosphorylase complexed with 5-FU. (**A**) The uridine phosphorylase complex (PDB ID 3NBQ). (**B**) The binding cavity. (**C**) The 5-FU binding mode.

Uridine phosphorylase plays a crucial role in the pyrimidine salvage pathway by facilitating the reversible phosphorolysis of uridine to uracil and ribose 1-phosphate [33]. This enzyme is widespread, found in prokaryotes, yeast, and higher organisms. The quest for potent and specific uridine phosphorylase inhibitors is driven by its modulating effect on the efficacy of pyrimidine nucleoside analogs, such as 5-FU, in chemotherapy [34]. Exogenous uridine administration can exert a protective effect against the toxic side effects of 5-FU chemotherapy (“uridine-rescue”) without compromising its anti-tumor efficacy. Given its pivotal role in 5-FU-based chemotherapy, uridine phosphorylase is an attractive target for drug development. Crystal structures of uridine phosphorylase complexed with 5-FU are available from various sources, including *Escherichia coli* (PDB ID 1RXC and 3KVV) [35,36], bovine *Bos taurus* (PDB ID 3KVR) [35], *Homo sapiens* (PDB ID 3NBQ) [37], *Salmonella typhimurium* (PDB ID 4E1V) [38], and *Schistosoma mansoni* (PDB ID 4TXN) [39]. The amino acid sequence of uridine phosphorylase is conserved across prokaryotes and eukaryotes (Table 4). The binding mode of uridine phosphorylases to 5-FU is similar, and for detailed analysis, we focus on the human enzyme (PDB ID 3NBQ). The binding of uracil is stabilized by a network of hydrogen bonds involving T141, Q217, R219, and a single deeply buried water molecule mediated by R94 (Figure 4). These amino acids are strictly conserved among known uridine phosphorylases, forming a uridine phosphorylase-specificity motif that distinguishes these enzymes with uridine preference from the larger family of nucleoside phosphorylases [40]. The fluorine moiety of 5-FU forms a hydrogen bond with G143 (3.8 Å) and is enclosed by a cluster of hydrophobic residues, including L272 (3.7 Å), L273 (3.5 Å), and I281 (3.5 Å). This comprehensive analysis of human uridine phosphorylase interactions with the therapeutic ligand 5-FU is crucial for the rational design of pharmacological inhibitors with potential medical applications.

### 3.5. rRNA N-Glycosidase

**Figure 5:**
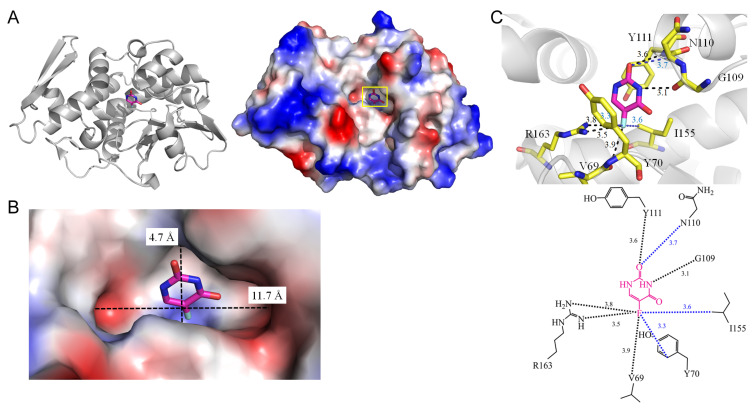
Crystal structure of rRNA N-glycosidase complexed with 5-FU. (**A**) The rRNA N-glycosidase complex (PDB ID 4O0O). (**B**) The binding cavity. (**C**) The 5-FU binding mode.

Ribosome-inactivating proteins (RIPs), characterized as N-glycosidases [41], are recognized for their ability to eliminate specific purine residues from the sarcin/ricin (S/R) loop of large rRNA [42]. This action leads to the inhibition of protein synthesis within the cell [43]. To comprehend the specific base recognition mechanism, the co-crystal structure of type 1 RIP (RIP1) from *Momordica balsamina* with pyrimidine [44] and 5-FU (PDB ID 4O0O) has been successfully determined. Key residues involved in the interaction with 5-FU include V69, Glu85, G109, N110, Y111, and R163 (Figure 5). The fluorine moiety of 5-FU forms hydrogen bonds with V69 (3.9 Å) and R163 (3.5 Å), contributing to the specific recognition of 5-FU. Considering that adenine-containing nucleosides/nucleotides serve as suitable substrates and the orientation of pyrimidine in the cleft differs from that of purine, it is suggested that pyrimidine-containing nucleosides/nucleotides could function as inhibitors. Consequently, the complex structure of RIP1 with 5-FU aids in understanding how pyrimidine-containing compounds may act as inhibitors of RIPs.

### 3.6. Uracil-DNA Glycosylase

**Figure 6:**
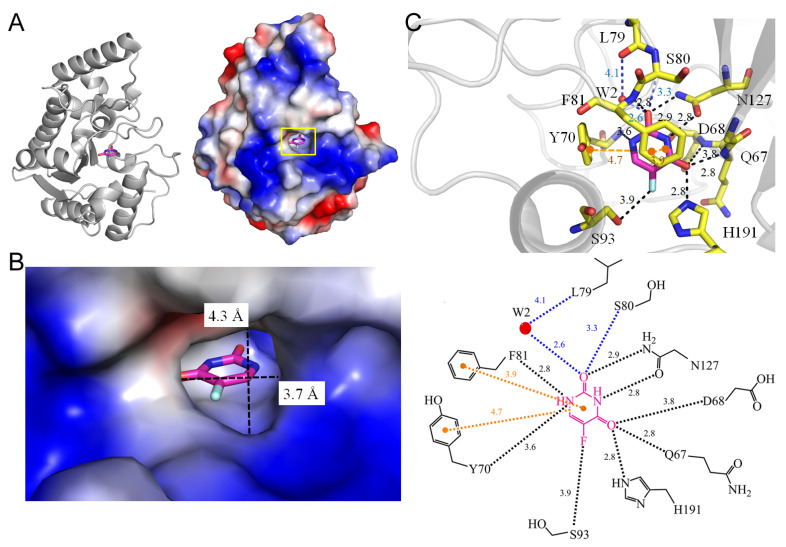
Crystal structure of uracil-DNA glycosylase complexed with 5-FU. (**A**) The uracil-DNA glycosylase complex (PDB ID 4WRY). (**B**) The binding cavity. (**C**) The 5-FU binding mode.

Uracil DNA glycosylase plays a crucial role in DNA repair by removing uracil from DNA through the cleavage of the glycosidic bond between uracil and deoxyribose [45]. Given that the spontaneous deamination of cytosine to uracil poses a mutagenic threat to organisms and can result in error-prone DNA replication, uracil-DNA glycosylase has evolved as a repair mechanism. Human uracil-DNA glycosylase, the prototypic and initially identified DNA glycosylase [46], is essential for removing deaminated cytosine as well as incorporated uracil and 5-FU from DNA [47]. These evolutionarily conserved DNA repair enzymes initiate the base excision repair pathway, emphasizing the potential of designing inhibitors against uracil-DNA glycosylase for treating various cancers [48] and infectious diseases [49]. The inhibitory effect of the uracil ring and its derivatives on *Mycobacterium tuberculosis* uracil-DNA glycosylase [50], achieved through specific and robust binding with the uracil-binding pocket, has been demonstrated. To further explore this strategy, the co-crystal structure of *M. tuberculosis* uracil-DNA glycosylase bound to 5-FU has been elucidated (PDB ID 4WRY) [51]. The binding of 5-FU is sustained by a network of hydrogen bonds involving Q67, D68, Y70, F81, S93, N127, H191, and a water molecule mediated by L79 (Figure 6). The π–π interactions with Y70 and F81 also take place for 5-FU. The fluorine moiety of 5-FU forms a hydrogen bond with S93. This complex structure with 5-FU, along with other pyrimidine analog complexes, provides a foundation for the design of structure-based inhibitors.

### 3.7. Pyrimidine Operon Regulatory Protein PyrR

**Figure 7:**
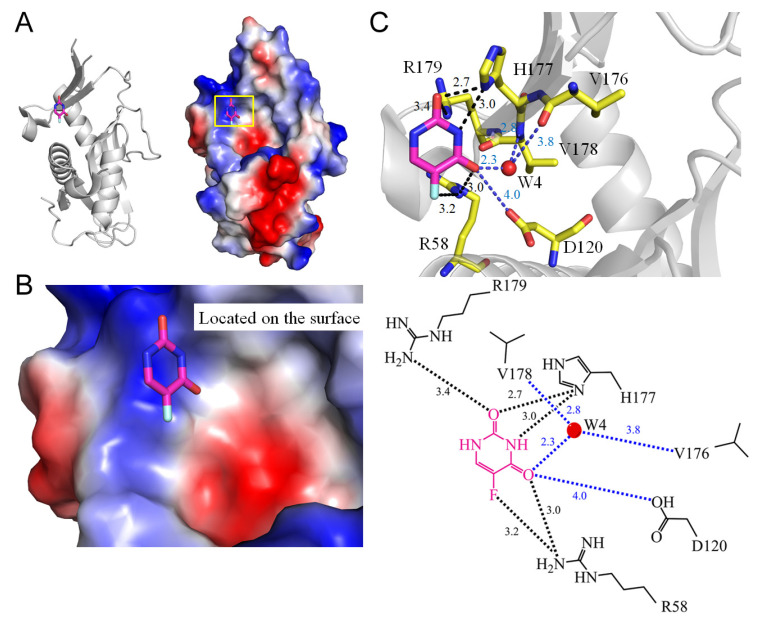
Crystal structure of PyrR complexed with 5-FU. (**A**) The PyrR complex (PDB ID 5IAO). (**B**) The binding cavity. (**C**) The 5-FU binding mode.

The pyrimidine operon regulatory protein (PyrR) functions as a regulator in de novo pyrimidine synthesis [52], positioned on the *pyr* operon alongside genes encoding enzymes for de novo pyrimidine biosynthesis. This pathway is pivotal in generating the preliminary nucleotides uridine 5′-monophosphate and uridine 5′-triphosphate for RNA synthesis. Elevated levels of these nucleotides trigger PyrR-mediated regulation, leading to transcription termination in the pathway [53]. PyrR accomplishes this by binding to the conserved mRNA sequence on the pyr operon, disrupting the anti-terminator [54]. Additionally, PyrR exhibits uracil phosphoribosyltransferase activity. In the presence of 5-FU, this enzyme activity facilitates the formation of fluorinated UMP, hindering DNA/RNA synthesis [9]. For a comprehensive understanding of the interactions between 5-FU and *Mycobacterium tuberculosis* PyrR, crucial for target-based anti-tuberculosis drug discovery, the co-crystal structure has been elucidated (PDB ID 5IAO) [55]. 5-FU forms hydrogen bonding contacts with R58, H177, and R179. V176 and V178 may also interact with 5-FU through water-molecule-mediated hydrogen bonding (Figure 7). This water molecule stabilizes the 5-FU interaction by forming hydrogen bonds with an oxygen atom from 5-FU and D120. The fluorine moiety of 5-FU establishes a hydrogen bond with R58 (3.2 Å), contributing to the specific recognition of 5-FU. Insights gained from these structural features can aid in understanding drug-resistance mechanisms and screening potential analogs with reduced 5-FU toxicity while maintaining effectiveness against *M. tuberculosis* [55].

### 3.8. PI3Kα

**Figure 8:**
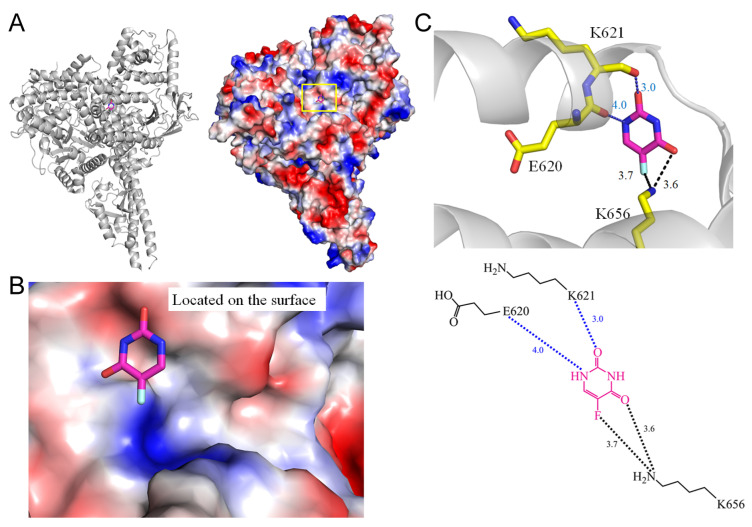
Crystal structure of PI3Kα complexed with 5-FU. (**A**) The PI3Kα complex (PDB ID 5SXC). (**B**) The binding cavity. (**C**) The 5-FU binding mode.

Phosphoinositide 3-kinases (PI3Ks), also known as phosphatidylinositol 3-kinases, constitute a family of enzymes integral to cellular functions such as cell growth, proliferation, differentiation, motility, survival, and intracellular trafficking [56,57]. The dysregulation of the PI3K/AKT/mTOR pathway is a common occurrence in various human cancers, including breast cancer, colorectal cancer, and hematologic malignancies. This underscores the significance of targeting this pathway as a potential therapeutic approach in cancer treatment. All PI3K isoforms play crucial roles in essential cellular processes such as metabolism, growth, proliferation, and migration. PI3Kα, in particular, is central to regulating glucose metabolism and growth, making it a promising target for anticancer drug development [58,59]. However, frequent mutations in the catalytic subunit of PI3Kα, observed in breast and other cancer types, pose limitations to chemotherapy. Understanding the complex structure of the PI3Kα mutant can provide insights into optimizing treatment efficacy while minimizing side effects [60]. To this end, the co-crystal structure of human PI3Kα bound to 5-FU has been elucidated (PDB ID 5SXC) [60]. The 5-FU binding site is located on the surface of the helical domain and, through structural analysis, residues E620 (4.0 Å), K621 (3.0 Å), and K656 (3.7 Å) are identified within contact distance, indicating interactions with 5-FU (Figure 8). The fluorine moiety of 5-FU forms a hydrogen bond with K656. This information may pave the way for the development of allosteric inhibitors for PI3K.

### 3.9. Dihydropyrimidinase

**Figure 9:**
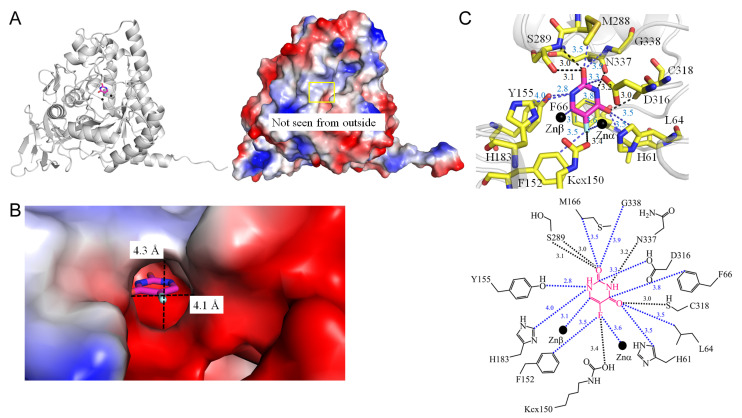
Crystal structure of dihydropyrimidinase complexed with 5-FU. (**A**) The dihydropyrimidinase complex (PDB ID 6KLK). (**B**) The binding cavity. (**C**) The 5-FU binding mode.

Dihydropyrimidinase [61] is ubiquitously found in living organisms, playing a crucial role in catalyzing a key step in the hydrolysis of dihydrouracil to N-carbamoyl-β-alanine during pyrimidine degradation [62,63]. As a member of the cyclic amidohydrolase family, which includes, dihydroorotase [64,65], and allantoinase [66,67,68] with similar active sites, dihydropyrimidinase features an unusual post-translational carbamylated modification of the Lys residue (Kcx) within its active site. Notably, dihydropyrimidinase exhibits the capacity to bind 5-FU [69] and 5-aminouracil [70]. Reports indicate 5-FU-associated toxicity in asymptomatic patients with dihydropyrimidinase deficiency undergoing anticancer therapy, leading to severe consequences, including fatalities [71]. The co-crystal structure of *Pseudomonas aeruginosa* dihydropyrimidinase with 5-FU has been elucidated (PDB ID 6KLK), providing insights into the various interactions between 5-FU and dihydropyrimidinase [69]. The binding of 5-FU is sustained by a network of hydrogen bonds involving S289 (3.0 Å), N337 (3.2 Å), C318 (2.9 Å), and Kcx150 (3.4 Å) (Figure 9). Residues Y155, H183, M166, G338, D316, H61, L64, F66, and F152 have been identified within contact distance, suggesting potential interactions with 5-FU. This structural information prompts further investigation to reassess the role of dihydropyrimidinase in anticancer and antipathogen therapy [72].

### 3.10. Dihydroorotases

**Figure 10:**
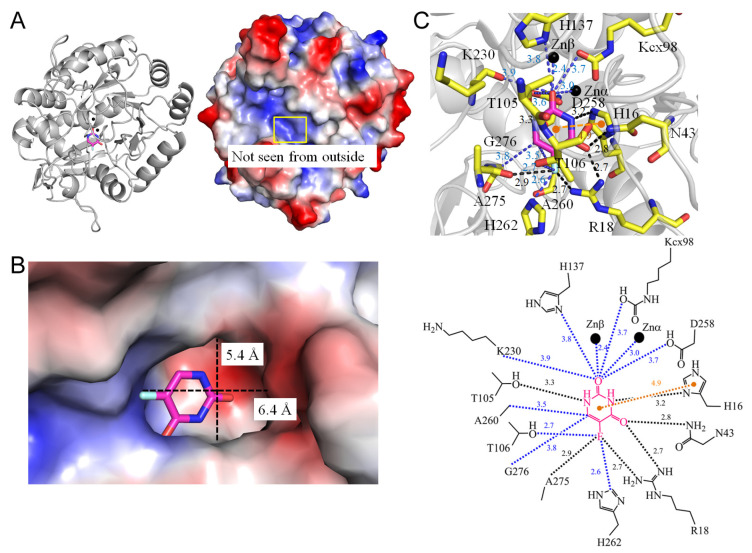
Crystal structure of the yeast dihydroorotase complexed with 5-FU. (**A**) The dihydroorotase complex (PDB ID 6L0B). (**B**) The binding cavity. (**C**) The 5-FU binding mode.

**Figure 11:**
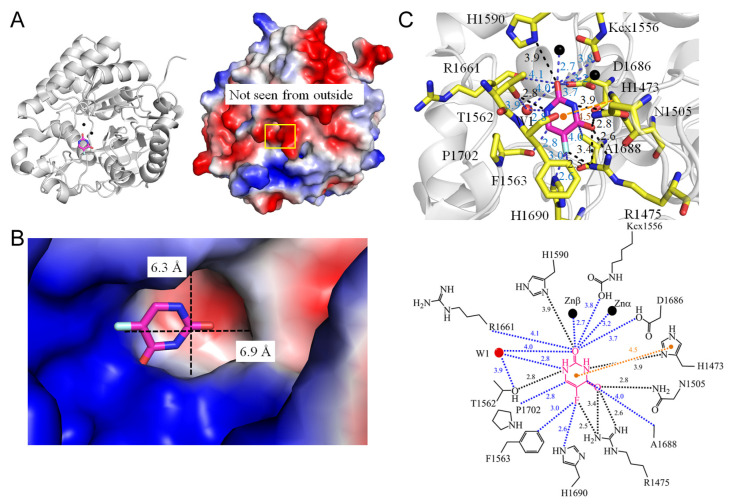
Crystal structure of the human dihydroorotase domain of CAD complexed with 5-FU. (**A**) The human dihydroorotase complex (PDB ID 8GVZ). (**B**) The binding cavity. (**C**) The 5-FU binding mode.

Dihydroorotase plays a pivotal role in catalyzing the cyclization of N-carbamoyl-L-aspartate to L-dihydroorotate, representing the third step in de novo pyrimidine biosynthesis [73]. While dihydroorotase activity is universally present in all organisms for the synthesis of pyrimidine nucleotides, phylogenetic and structural analyses have unveiled at least three distinct forms of dihydroorotase [74]. In mammals, dihydroorotase is part of a single trifunctional polypeptide of 240 kDa (CAD), alongside two other enzymes—carbamoyl phosphate synthetase and aspartate transcarbamoylase [75]. This trifunctional complex self-assembles into a hexamer of 1.5 MDa. In yeasts, dihydroorotase exists as a monofunctional protein [74]. The structural disparities among dihydroorotases make them attractive targets for pharmacological inhibition, potentially impacting cancer cells, malarial parasites, and rapidly growing pathogens [75]. In analyzing the complexed crystal structure of *Saccharomyces cerevisiae* dihydroorotase (PDB ID 6L0B) [76], it is evident that the binding of 5-FU relies on a network of hydrogen bonds involving residues H16, R18, N43, T105, and A275 (Figure 10). The π–π interaction with H16 also takes place for 5-FU. Notably, R18 (2.7 Å) and A275 (2.9 Å) exhibit specific interactions with the fluorine moiety of 5-FU. T106 (2.7 Å) and H162 (2.6 Å), within contact distance, also contribute to this recognition of the fluorine moiety of 5-FU. Examining the co-crystal structure reveals how the dihydroorotase domain in human CAD binds to 5-FU (PDB ID 8GVZ) [77]. In this context, the binding of 5-FU involves a network of hydrogen bonds with human enzyme residues H1473, R1475, N1505, T1562, and H1590 (Figure 11). Notably, R1475 (2.6 Å) exhibits specific recognition with the fluorine moiety of 5-FU. T1562 interacts with 5-FU through water-molecule-mediated hydrogen bonding. The π–π interaction with H1473 also takes place for 5-FU. Additionally, F1563 (3.0 Å) and H1690 (2.6 Å), within contact distance, contribute to the specific recognition of the fluorine moiety of 5-FU. These structural insights suggest distinct binding modes for 5-FU in these two dihydroorotases, offering a potential strategy for designing anticancer drugs specifically targeting the human dihydroorotase.

### 3.11. Single-Stranded DNA-Binding Proteins SsbA and SsbB

**Figure 12:**
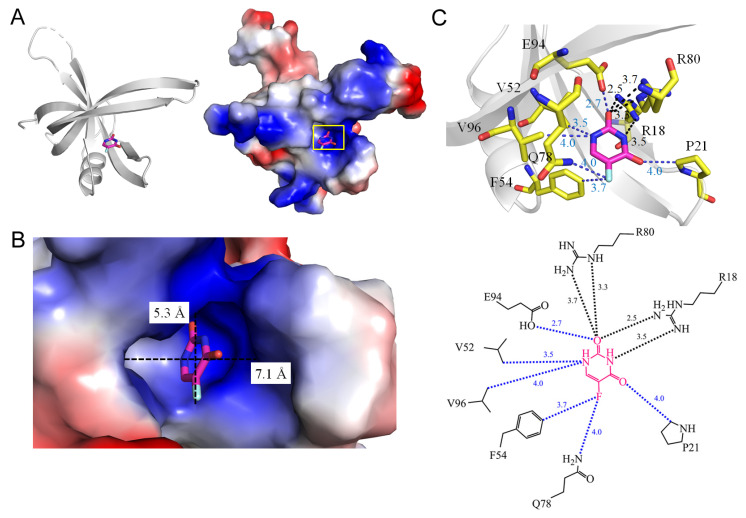
Crystal structure of SsbA complexed with 5-FU. (**A**) The SsbA complex (PDB ID 7YM1). (**B**) The binding cavity. (**C**) The 5-FU binding mode.

**Figure 13:**
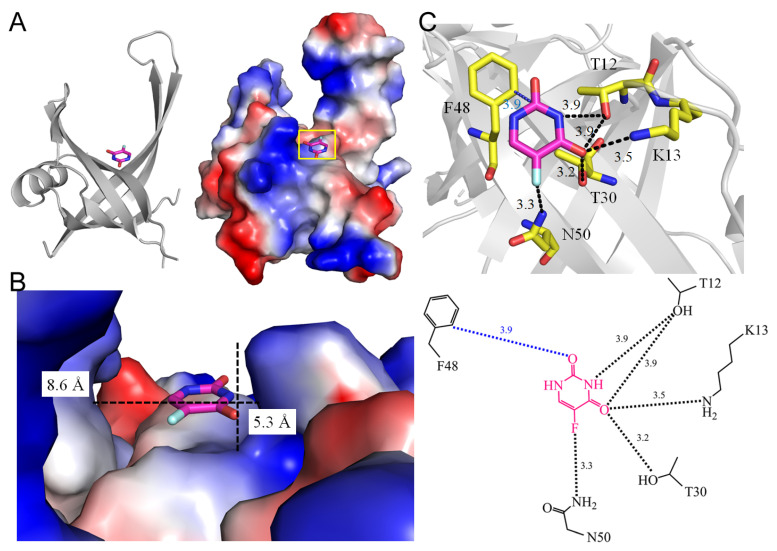
Crystal structure of SsbB complexed with 5-FU. (**A**) The SsbB complex (PDB ID 7DEP). (**B**) The binding cavity. (**C**) The 5-FU binding mode.

Single-stranded DNA-binding proteins (SSBs) play a pivotal role in all DNA-dependent cellular processes [78], and are highly conserved across organisms, underscoring their fundamental importance [78,79,80]. Their multifaceted functions in DNA replication, recombination, repair, and other cellular processes position SSBs as critical guardians of genomic integrity [81]. By binding specifically to single-stranded DNA (ssDNA) with high affinity, SSBs prevent re-annealing, shield DNA from nucleases, and facilitate accessibility to other DNA-binding proteins [82]. Bacterial SSBs have been extensively studied [83,84,85,86], typically adopting homotetrameric configurations with four oligonucleotide/oligosaccharide-binding folds (OB-folds) for ssDNA binding [87,88,89,90]. PriB, a distinct SSB variant with two OB-folds, exhibits a unique ssDNA binding mode [91,92,93]. Exploring the structures of SSBs provides molecular insights relevant to antipathogen chemotherapy [94,95,96,97,98,99]. While *E. coli* possesses a single SSB (EcSSB), *Staphylococcus aureus* [100] has three paralogous SSBs: SsbA [101], SsbB [102], and SsbC [103]. SsbA, with sequence similarity to EcSSB, and SsbB, implicated in chromosome segregation [104] and transformation [105], have been identified as 5-FU binders. The function of SsbC remains undetermined. The co-crystal structures of SsbA (PDB ID 7YM1) [106] and SsbB (PDB ID 7DEP) [107] bound to 5-FU reveal key residues involved in binding, such as R18 (2.5 Å), P21 (4.0 Å), V52 (3.5 Å), F54 (3.7 Å), Q78 (4.0 Å), R80 (3.3 Å), E94 (2.7 Å), and V96 (4.0 Å) in SsbA (Figure 12) and T12 (3.9 Å), K13 (3.5 Å), T30 (3.2 Å), F48 (3.9 Å), and N50 (3.3 Å) in SsbB (Figure 13). Notably, F54 and Q78 in SsbA, and N50 in SsbB, within contact distance, contribute to the specific recognition of the fluorine moiety of 5-FU. Despite the high sequence and structural similarities between SsbA and SsbB, their 5-FU-complexed structures unveil distinct configurations, highlighting unique 5-FU binding sites. These structural analyses elucidate the mechanisms governing the recognition of different 5-FU binding sites, even in proteins with similar sequences and structures. Thus, not only the interacting residues, but also the variation in the binding groove width may potentially influence the mechanisms governing 5-FU binding between SsbA and SsbB [106].

### 3.12. Hydroxydechloroatrazine Ethylaminohydrolase VCZ

**Figure 14:**
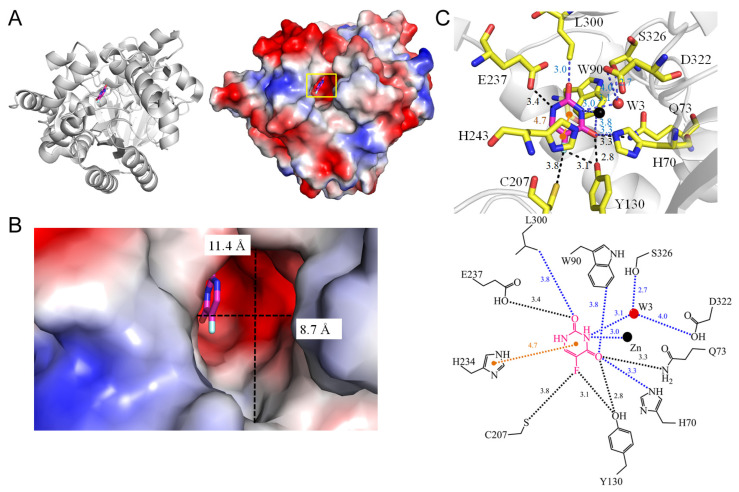
Crystal structure of VCZ complexed with 5-FU. (**A**) The VCZ complex (PDB ID 8IS4). (**B**) The binding cavity. (**C**) The 5-FU binding mode.

Cytosine deaminase, a member of the amidohydrolase superfamily, catalyzes the conversion of cytosine to uracil [108]. Predominantly present in bacteria and fungi, this enzyme is absent in mammalian cells [109], making it an ideal candidate for converting the low-toxic prodrug, 5-fluorocytosine, to the cytotoxic product 5-FU [110]. However, the presence of intestinal flora cytosine deaminase can lead to the undesired production of 5-FU from 5-fluorocytosine, limiting its application in anticancer chemotherapy and causing detrimental effects on the organism [111]. To address this issue, hydroxydechloroatrazine ethylaminohydrolase (isocytosine specific deaminase VCZ) from *Obesumbacterium proteus* comes into play. VCZ can specifically convert isocytosine (the isomer of cytosine) and 5-fluoroisocytosine to uracil and 5-FU, providing an alternative enzyme/prodrug system for cancer therapy [112]. Cytosine is not a substrate of VCZ. To delve deeper into the catalytic relationship between VCZ and the product 5-FU, their co-crystal structure has been elucidated (PDB ID 8IS4) [113]. Within this complex structure, both VCZ protomers exhibit a 5-FU molecule with an identical binding environment. The binding of 5-FU is facilitated by a network of hydrogen bonds involving Q73, E237, Y130, and C207, along with a water molecule mediated by D322 and S326 (Figure 14). The π–π interaction with H234 takes place for 5-FU. The fluorine moiety of 5-FU forms hydrogen bonds with Y130 (3.1 Å) and C207 (3.8 Å). This detailed complex structure with 5-FU contributes valuable insights into understanding the catalytic specificity of VCZ, paving the way for further advancements in gene-directed enzyme prodrug therapy (GDEPT) [114].

## 4. Interaction Patterns

**Figure 15:**
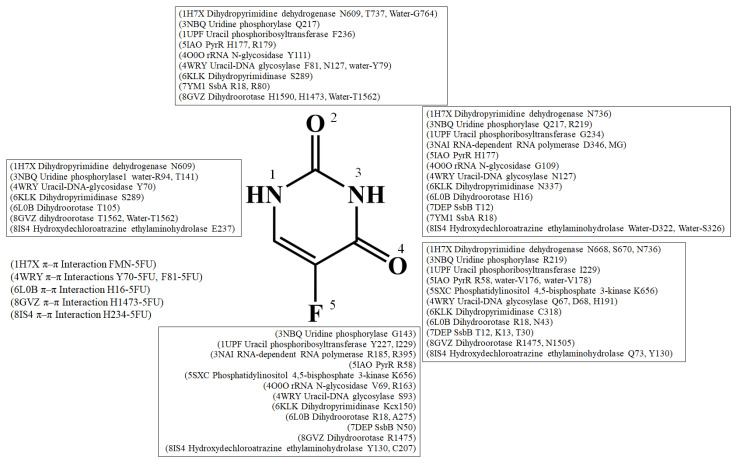
The interactions of 5-FU with the residues analyzed using the complexed structures available in the PDB.

5-FU stands as one of the most widely prescribed chemotherapeutics, administered to approximately 2 million individuals globally each year, either as 5-FU itself or in the form of its prodrugs (e.g., capecitabine). Despite more than 60 years of study [115,116], a systematic review of how 5-FU binds to proteins has been lacking. Investigating the specific binding patterns of 5-FU to proteins is essential for identifying additional interacting proteins and comprehending their medical implications. An analysis of the 5-FU binding environment was conducted based on available complex structures (Figure 15 and Table 3). Two groups of residues emerged upon 5-FU binding, classified as P- and R-type residues. These high-frequency interactive residues with 5-FU include positively charged residues Arg and Lys, grouped as the P type, and ring residues Phe, Tyr, Trp, and His, grouped as the R type. Due to their high occurrence, the 5-FU binding modes were simplistically classified into three types, based on a distance criterion (4 Å) to discriminate between interacting and non-interacting residues around the ligand 5-FU (Table 5):

1. Type 1 (P-R type): The contact distance involves residues from both P (Arg and Lys) and R (Phe, Tyr, Trp, and His) types.
2. Type 2 (P type): The contact distance involves more than two P-type residues, Arg and/or Lys.
3. Type 3 (R type): The contact distance involves R-type residues, Phe, Tyr, Trp, and/or His.

**Table 5:** Summary of the interactions of 5-FU with the residues.

Type	PDB	Interactive Residues	HB	WB	π-π Int.	F5 (HB)	F5 (CD)	N1 or N3 (HB)	N1 or N3 (CD)	N3 (HB)	N3 (CD)	O4 (HB)	O4 (CD)
1	3NBQ	**R94**, T141, S142, G143, **F213**, Q217, **R219**, I247, E248, M249, L272, L273, I281, W1-**R94**(B)	T141, G143, Q217, **R219**	W1-**R94**(B)	-	G143 [N]	I281, L272, L273	T141 [OG1], Q217 [OE1], **R219** [NH1]	**F213**	Q217 [OE1], **R219** [NH1]	**F213**	**R219** [NH1] [NH2]	-
1	4O0O	V69, **Y70**, G109, N110, **Y111**, I155, **R163**	V69, G109, **Y111**, **R163**	-	-	V69 [O], **R163** [NH1], **R163** [NH2]	I155,**Y70**	G109 [O]	-	G109 [O]	-	-	-
1	5IAO	**R58**, D120, V176, **H177**, **R179**, W4-V178	**R58**, **H177**, **R179**,	W4-V178	-	**R58** [NH1], **R58** [NE2]	D120	**H177** [ND1]	-	**H177** [ND1]	-	**R58** [NH1]	D120
1	6KLK	**H61**, L64, **F66**, **Kcx150**, **F152**, **Y155**, **H183**, M288, S289, D316, C318, N337, G338,	**Kcx150**, S289, D316, C318, N337, G338,	-	-	**Kcx150** [OQ2]	**F152**	S289 [O], N337 [O]	**Y155**, **H183**	N337 [O]		C318 [SG]	**H61**, L64
1	6L0B	**H16**, **R18**, N43, **Kcx98**, T105, T106, **H137**, **K230**, D258, A260, **H262**, G276, A275	**H16**, **R18**, N43, T105	-	**H16** (4.9Å)	A275 [O]	**H262**	T105 [OG1], **H16** [ND1]	-	**H16** [ND1]	-	**R18** [NH1], N43 [ND2]	-
1	7DEP	T12, **K13**, T30, **F48**, N50	T12, **K13**, T30, N50	-	-	N50 [ND2]	-	T12 [OG1],	-	T12 [OG1],	-	T12 [OG1], **K13** [NZ], T30 [OG1]	-
1	7YM1	**R18**, P21, **F54**, V52, Q78, **R80**, E94, V96	**R18**, **R80**	-	-		Q78, **F54**	**R18** [NH2]	V52, V96	**R18** [NH2]	-	-	P21
1	8GVZ	**R1475**, **H1473**, N1505, **Kcx1556**, T1562, **F1563**, **H1590**, **R1661**, D1686, A1688, **H1690**, P1702,	**R1475**, **H1473**, N1505, T1562, **H1590**	W1- T1562	**H1473** (4.5 Å)	**R1475** [NH2]	**F1360**, **H1690**	T1562 [OG1], **H1473** [ND1]	-	**H1473** [ND1]	-	**R1475** [NH1] [NH2], N1505 [ND2]	-
2	3NAI	**R185**, D245, **R395**, D346, D347	**R185**, D245, **R395**	-	-	**R185** [NH1], **R185** [NH2], **R395** [NH2]	-	D346 [OD2]	-	D346 [OD2]	-	-	-
2	5SXC	E620, **K621**, **K656**	**K656**	-	-	**K656** [NZ]	-	-	E620	-	-	**K656** [NZ]	-
3	1H7X	N609, E611, L612, I613, N668, S670, N736, T737, W2-G764	N609, N668, S670, N736, T737	W2-G764	-	-	L612, I613, **FMN**	N609 [OD1], N736 [OD1]	-	N609 [OD1], N736 [OD1]	-	N668 [ND2], S670 [OG], N736 [ND2]	-
3	1UPF	M166, A168, **Y227**, **Y228**, I229, G234, **F236**, D316	**Y227**, I229, G234, **F236**	-	-	**Y227** [O], I229 [N]	A168	G234 [O]	-	G234 [O]	-	I229 [N]	-
3	4WRY	G66, Q67, D68, **Y70**, S80, **F81**, S93, N127, **H191**, W2-L79	G66, Q67, D68, **Y70**, S93, N127, **H191**	W2-L79	**Y70** (4.7 Å) **F81** (3.9 Å)	S93 [OG]	-	**Y70** [N], N127 [OD1]	-	N127 [OD1]	-	Q67 [N], D68 [N], **H191** [NE2]	-
3	8IS4	**H70**, Q73, **W90**, **Y130**, C207, E237, L300, D322, W3-S326	Q73, **Y130**, C207, E237	W3-S326	**H234** (4.7 Å)	C207 [SG], **Y130** [OH]	-	-	-	-	-	Q73 [NE2], **Y130** [OH]	**W90** [CH2] **H70** [NE2]

The residues from P (black bold) and R (blue bold) types are highlighted. HB, hydrogen bond; WB, water bridge; CD, residues within the contact distance.

In the complex structure of dihydropyrimidine dehydrogenase (Figure 1), 5-FU is stacked with FMN, indicating a Type 3 binding pattern. For instance, in the uridine phosphorylase complex, R219 (P-type residue) and F231 (A-type residue) interact with 5-FU within the contact distance (<4 Å), suggesting a Type 1 binding pattern (see Table 3 and Table 5). In the RNA-dependent RNA polymerase complex, R185 and R395 (P-type residues) interact with 5-FU within the contact distance (<4 Å), indicating a Type 2 binding pattern. In the uracil phosphoribosyltransferase complex, F236 and Y227 (A-type residues) interact with 5-FU, suggesting a Type 3 binding pattern. In summary, among these 14 complex structures, 8 conform to the Type 1 pattern, 2 conform to the Type 2 pattern, and 4 conform to the Type 3 pattern.

We also examined residues with high interaction frequencies involving the N1, N3, O4, and F5 atoms of 5-FU (Table 6). When a P- or R-type residue is within the contact distance (<4 Å) of the fluorine moiety (F5) of 5-FU, the binding incidence is 78.6% (11/14). Expanding to N3, O4, or F5 with a P- or R-type residue within the contact distance results in a binding incidence of 100%, suggesting that the amide N3-C-O4 and F5 constitute crucial recognition sites for proteins. Additionally, among the 14 classes of structures, 5 contain metal cofactors, all of which interact with 5-FU (Table 7). This strongly suggests that the presence of metal in any protein should be considered an important binding factor for 5-FU. The dimensions (x, y) of the protein cavity also appear to be a critical factor for the binding of 5-FU. Any one dimension < 11 Å corresponds to a 5-FU binding incidence of 64.3%, while dimensions < 12 Å result in an incidence of 92.9%. These findings, derived from the correlation in these 14 structural pieces of evidence, suggest that the small molecule 5-FU may still preferentially access suitable binding sites for stable complex formation.

## 5. Conclusions

This review not only reports on published work, but also combines it with our analysis (critical review). In this way, it presents a comprehensive summary of the binding modes observed for 5-FU. Collectively, these interaction analyses contribute to the construction of a structural interactome delineating the associations of the anticancer drug 5-FU. The findings offer a structural perspective on the specific binding patterns of 5-FU within protein pockets or on the protein surface. In the quest for identifying novel 5-FU binding proteins, it is highlighted that proteins featuring an appropriate pocket size (with a dimension of <12 Å) and the presence of P/R-type residues within the contact distance represent highly possible sites for interactions with 5-FU.

## Figures and Tables

**Table 1:** Crystal structures of 5-FU complexes in PDB.

PDB ID	Organism	Crystal Structure	Unique Ligand
1H7X	*Sus scrofa*	Dihydropyrimidine dehydrogenase (DPD) from pig, ternary complex of a mutant enzyme (C671A), NADPH and 5-fluorouracil	FAD, FMN, NDP, SF_4_, URF
1RXC	*Escherichia coli* (strain K12)	*E. coli* uridine phosphorylase: 5-fluorouracil ribose-1-phosphate complex	5UD, K, PO_4_, R1P, URF
1UPF	*Toxoplasma gondii*	Structure of the uracil phosphoribosyltransferase, mutant C128V bound to the drug 5-fluorouracil	SO_4_, URF
3KVR	*Bos taurus*	Trapping of an oxocarbenium ion intermediate in UP crystals	R2G, SO_4_, URF
3KVV	*Escherichia coli* (strain K12)	Trapping of an oxocarbenium ion intermediate in UP crystals	R2B, SO_4_, URF
3NAI	Murine norovirus 1	Crystal structures and functional analysis of murine norovirus RNA-dependent RNA polymerase	GOL, MG, MN3, SO_4_, URF
3NBQ	*Homo sapiens*	Human uridine phosphorylase 1 (hUPP1) with 5-fluorouracil	URF
4E1V	*Salmonella typhimurium*	X-RAY structure of the uridine phosphorylase from *Salmonella typhimurium* in complex with 5-fluorouracil at 2.15 Å resolution	EDO, GOL, K, URF
4O0O	*Momordica balsamina*	Crystal structure of the complex of type 1 ribosome inactivating protein from *Momordica balsamina* with 5-fluorouracil at 2.59 Å resolution	GOL, NAG, URF
4TXN	*Schistosoma mansoni*	Crystal structure of uridine phosphorylase from *Schistosoma mansoni* in complex with 5-fluorouracil	SO_4_, URF
4WRY	*Mycobacterium tuberculosis*	Crystal structure of *Mycobacterium tuberculosis* uracil-DNA glycosylase in complex with 5-fluorouracil (B), Form I	CIT, CL, URF
4WRZ	*Mycobacterium tuberculosis*	Crystal structure of *Mycobacterium tuberculosis* uracil-DNA glycosylase in complex with 5-fluorouracil (AB), Form I	CIT, CL, IPA, URF
4WS0	*Mycobacterium tuberculosis*	Crystal structure of *Mycobacterium tuberculosis* uracil-DNA glycosylase in complex with 5-fluorouracil (A), Form II	CL, EDO, URF
4WS1	*Mycobacterium tuberculosis*	Crystal structure of *Mycobacterium tuberculosis* uracil-DNA glycosylase in complex with 5-fluorouracil (AB), Form II	CL, EDO, URF
5IAO	*Mycobacterium tuberculosis*	Structure and mapping of spontaneous mutational sites of PyrR from *Mycobacterium tuberculosis*	URF
5SXC	*Homo sapiens*	Crystal structure of PI3Kalpha in complex with fragment 8	SEP, URF
6KLK	*Pseudomonas aeruginosa*	Crystal structure of the *Pseudomonas aeruginosa* dihydropyrimidinase complexed with 5-FU	KCX, URF, ZN
6L0B	*Saccharomyces cerevisiae*	Crystal structure of dihydroorotase in complex with fluorouracil from *Saccharomyces cerevisiae*	KCX, URF, ZN
7D8J	*Staphylococcus aureus*	*S. aureus* SsbB with 5-FU	URF
7DEP	*Staphylococcus aureus*	*S. aureus* SsbB with 5-FU	URF
7YM1	*Staphylococcus aureus*	Structure of SsbA protein in complex with the anticancer drug 5-fluorouracil	GOL, URF
8GVZ	*Homo sapiens*	Crystal structure of the human dihydroorotase domain in complex with the anticancer drug 5-fluorouracil	KCX, URF, ZN
8IS4	*Obesumbacterium proteus*	Structure of an isocytosine specific deaminase VCZ in complex with 5-FU	GOL, TRS, URF, ZN

In this article, structures complexed with 5-FU, namely PDB IDs 1H7X, 1UPF, 3NAI, 3NBQ, 4O0O, 4WRY, 5IAO, 5SXC, 6KLK, 6L0B, 7DEP, 7YM1, 8GVZ, and 8IS4 are analyzed to avoid redundancy.

**Table 2:** The sizes of the 5-FU binding sites.

Molecule	Type of Binding Site	Area *	Volume *	Dimensions (x, y) of the Cavity (Å) #
Dihydropyrimidine dehydrogenase	Pocket	59.2	23.12	4.8, 7.1
Uridine phosphorylase	Pocket	138.5	83.4	2.8, 6.7
Uracil phosphoribosyltransferase	Pocket	566	710	11.5, 8.7
RNA-dependent RNA polymerase	Pocket	2976	4400	17.3, 5.3
PyrR	Surface	ND	ND	ND
Phosphatidylinositol 4,5-bisphosphate 3-kinase	Surface	ND	ND	ND
rRNA N-glycosidase	Pocket	82.6	39.4	11.7, 4.7
Uracil-DNA glycosylase	Pocket	278.9	169.9	3.7, 4.3
Dihydropyrimidinase	Pocket	132.6	45.9	4.1, 4.3
Dihydroorotase (yeast)	Pocket	144.0	44.8	6.4, 5.4
SsbB	Pocket	31.1	8.8	5.3, 8.6
SsbA	Pocket	369	452	7.1, 5.3
Dihydroorotase (human)	Pocket	289.7	213.4	6.9, 6.3
Hydroxydechloroatrazine ethylaminohydrolase	Pocket	544	437	8.7, 11.4

* The value was calculated via CASTp 3.0 [20]. # The value was manually measured via PyMol. ND, not determine.

**Table 3:** The 5-FU interactions.

Hydrogen Bond			The Contact Distance within <4 Å		
5-FU	Dist. [Å]	Residue	5-FU	Dist. [Å]	Residue
Type 1 (P-R type)					
1.1 Uridine phosphorylase from *Homo sapiens* (3NBQ)					
5FU [N1]	4.1	T141 [OG1] #	5FU [O2]	3.6	E248 [CA]
5FU [N1]/W1	3.8/4.0	R94 [NH2] #	5FU [O2]	3.6	M249 [CG]
5FU [O2]	2.9	Q217 [NE2]	5FU [C2]	3.8	I247 [O]
5FU [N3]	2.7	Q217 [OE1]	5FU [N3]	3.4	F231 [CE1]
5FU [N3]	4.0	R219 [NH1]	5FU [F5]	3.7	L272 [CD1]
5FU [O4]	2.9	R219 [NH2]	5FU [F5]	3.5	L273 [CD2]
5FU [O4]	3.2	R219 [NH1] *	5FU [F5]	3.5	I281 [CD1]
5FU [F5]	3.8	G143 [N] #			
1.2 rRNA N-glycosidase from *Momordica balsamina* (4O0O)					
5FU [O2]	3.6	Y111 [N]	5FU [O2]	4.0	N110 [O2]
5FU [N3]	3.1	G109 [O]			
5FU [F5]	3.9	V69 [O]			
5FU [F5]	3.8	R163 [NH1] *			
5FU [F5]	3.5	R163 [NH2] *			
1.3 PyrR from *Mycobacterium tuberculosis* (5IAO)					
5FU [O2]	2.7	H177 [ND1]	5FU [O4]	4.0	D120 [O4]
5FU [O2]	3.4	R179 [NH2] #			
5FU [N3]	3.0	H177 [ND1] #			
5FU [O4]	3.0	R58 [NH1]			
5FU [O4]/W4	2.3/3.8	V176 [O] #			
5FU [O4]/W4	2.3/2.8	V178 [N] #			
5FU [F5]	3.2	R58 [NH1] *			
1.4 Dihydropyrimidinase from *Pseudomonas aeruginosa* (6KLK)					
5FU [N1]	3.1	S289 [O] #	5FU [N1]	2.8	Y155 [OH]
5FU [O2]	3.0	S289 [N] *	5FU [N1]	4.0	H183 [CE1]
5FU [N3]	3.2	N337 [O] #	5FU [O2]	3.5	M166 [CB]
5FU [O4]	2.9	C318 [SG] *	5FU [O2]	3.9	G338 [N]
5FU [F5]	3.4	Kcx150 [OQ2] *	5FU [C2]	3.8	D316 [OD2]
			5FU [O2]	3.5	H61 [CD2]
			5FU [O2]	3.5	L64 [CD2]
			5FU [C4]	3.8	F66 [CE2]
			5FU [F5]	3.5	F152 [CE1]
1.5 Dihydroorotase from *Saccharomyces cerevisiae* (6L0B)					
5FU [N1]	3.3	T105 [OG1] #	5FU [O2]	3.7	Kcx98 [OQ2]
5FU [N3]	3.2	H16 [ND1]	5FU [O2]	3.8	H137 [ND1]
5FU [O4]	2.7	R18 [NH1]	5FU [O2]	3.9	K230 [O]
5FU [O4]	2.8	N43 [ND2]	5FU [O2]	3.7	D258 [OD2]
5FU [F5]	2.8	R18 [NH2]	5FU [F5]	2.7	T106 [OG]
5FU [F5]	2.9	A275 [O] *	5FU [F5]	2.6	H162 [NE2]
			5FU [C6]	3.5	A260 [CB]
			5FU [C6]	3.8	G276 [CA]
1.6 SsbB from *Staphylococcus aureus* (7DEP)					
5FU [N3]	3.9	T12 [OG1] #	5FU [O2]	3.9	F48 [CD2]
5FU [O4]	3.9	T12 [OG1] #			
5FU [O4]	3.5	K13 [NZ]			
5FU [O4]	3.2	T30 [OG1] *			
5FU [F5]	3.3	N50 [ND2] *			
1.7 SsbA from *Staphylococcus aureus* (7YM1)					
5FU [O2]	3.5	R18 [NH1] #	5FU [N1]	3.5	V52 [CG1]
5FU [O2]	3.7	R80 [NH2] #	5FU [N1]	4.0	V96 [CG1]
5FU [O2]	3.3	R80 [NE]	5FU [O2]	2.7	E94 [OE1]
5FU [N3]	2.5	R18 [NH2]	5FU [O4]	4.0	P21 [CD]
			5FU [F5]	3.7	F54 [CZ]
			5FU [F5]	4.0	Q78 [NE2]
1.8 Dihydroorotase from *Homo sapiens* (8GVZ)					
5FU [N1]	2.9	T1562 [OG1] #	5FU [O2]	3.8	K1556 [OQ1]
5FU [N1]/W1	3.9/2.8	T1562 [OG1] #	5FU [O2]	4.1	R1661 [O]
5FU [O2]	3.9	H1590 [ND1]	5FU [O2]	3.7	D1686 [OD2]
5FU [O2]/W1	3.9/4.0	T1562 [OG1] #	5FU [C4]	4.0	A1688
5FU [N3]	3.9	H1473 [ND1] #	5FU [F5]	3.0	F1563 [CD2]
5FU [O4]	2.6	R1475 [NH1]	5FU [F5]	2.6	H1690 [CE1]
5FU [O4]	3.4	R1475 [NH2] #	5FU [C6]	2.8	P1702 [O]
5FU [O4]	2.8	N1505 [ND2]			
5FU [F5]	2.6	R1475 [NH2] *			
Type 2 (P type)					
2.1 RNA-dependent RNA polymerase from murine norovirus 1 (3NAI)					
5FU [N3]	2.4	D346 [OD2] *	5FU [O2]	3.2	D245 [OD11]
5FU [O4]	4.0	R185 [NH1] #	5FU [O2]	2.6	D347 [OD2]
5FU [F5]	3.7	R185 [NH1] *			
5FU [F5]	3.6	R185 [NH2]			
5FU [F5]	3.4	R395 [NH2] *			
2.2 Phosphatidylinositol 4,5-bisphosphate 3-kinase from *Homo sapiens* (5SXC)					
5FU [O4]	3.6	K656 [NZ]	5FU [N1]	4.0	E620 [O]
5FU [F5]	3.7	K656 [NZ] *	5FU [O2]	3.0	K621 [O]
Type 3 (R type)					
3.1 Dihydropyrimidine dehydrogenase from *Sus scrofa* (1H7X)					
5FU [N1]	2.9	N609 [OD1]	5FU [F5]	3.6	L162 [C]
5FU [O2]	2.9	N609 [ND2]	5FU [F5]	3.8	I163 [CG2]
5FU [O2]	2.9	T737 [OG1]	5FU [C6]	3.0	E611 [O]
5FU [O2]/W2	3.5/3.2	G764 [N] #			
5FU [N3]	3.0	N736 [OD1]			
5FU [O4]	3.1	N668 [ND2]			
5FU [O4]	3.3	S670 [OG]			
5FU [O4]	3.0	N736 [ND2]			
3.2 Uracil phosphoribosyltransferase from *Toxoplasma gondii* (1UPF)					
5FU [O2]	3.6	F236 [N]	5FU [O2]	3.2	D235 [OD1]
5FU [N3]	3.2	G234 [O]	5FU [N3]	3.8	M166 [CG]
5FU [O4]	3.4	I229 [N]	5FU [C4]	3.8	T228 [CD2]
5FU [F5]	3.8	I229 [N] *	5FU [F5]	3.1	A168 [CB]
5FU [F5]	3.0	Y227 [O]			
3.3 Uracil-DNA glycosylase from *Mycobacterium tuberculosis* (4WRY)					
5FU [N1]	3.6	Y70 [N]	5FU [O2]	3.3	S80 [CA]
5FU [O2]	2.9	N127 [ND2]			
5FU [O2]	2.8	F81 [N]			
5FU [O2]/W2	2.6/4.1	L79 [O] #			
5FU [N3]	2.8	N127 [OD1]			
5FU [O4]	2.8	Q67 [N]			
5FU [O4]	3.8	D68 [N]			
5FU [O4]	2.8	H191 [NE2] *			
5FU [F5]	3.9	S93 [OG] *			
3.4 Hydroxydechloroatrazine ethylaminohydrolase from *Obesumbacterium proteus* (8IS4)					
5FU [N1]	3.4	E237 [OE1]	5FU [O2]	3.0	L300 [CD1]
5FU [N3]/W3	3.1/4.0	D322 [OG1] #	5FU [O4]	3.8	W90 [CH2]
5FU [N3]/W3	3.1/2.7	S326 [OG] #	5FU [O4]	3.3	H70 [NE2]
5FU [O4]	3.3	Q73 [NE2]			
5FU [O4]	2.7	Y130 [OH]			
5FU [F5]	3.1	Y130 [OH]			
5FU [F5]	3.8	C207 [SG] *			

π–π interactions were predicted through PLIP: Y70 (4.7 Å) and F81 (3.9 Å) in PDB 4WRY; H16 (4.9 Å) in PDB 6L0B; H1473 (4.5 Å) in PDB 8GVZ; H234 (4.7 Å) in PDB 8IS4. * The hydrogen bonds were predictable only via PISA. # The hydrogen bonds were predictable only via PLIP.

**Table 4:** Sequence similarity of uridine phosphorylases.

PDB ID	Organism	Length	Identities (%)	Positives (%)
3NBQ	*Homo sapiens*	310	100	100
4TXN	*Schistosoma mansoni*	296	44	64
1RXC	*Escherichia coli* (strain K12)	253	32	46
4ETV	*Salmonella typhimurium*	253	27	41

**Table 6:** The frequency of 5-FU binding in proximity to P- or R-type residues.

Binding Type	Number	Frequency (%)	F5	N1 or N3	N3	O4	N1, N3, or F5	N3, O4, or F5	Binding Pocket	One Dimension < 11 Å	One Dimension < 12 Å
Type 1 (P-R type)	8	8/14 (57.1%)	6/8 (75%)	6/8 (75%)	5/8 (35.7%)	6/8 (75%)	7/8 (87.5%)	8/8 (100%)	7/8 (87.5%)	7/8 (87.5%)	8/8 (100%)
Type 2 (P type)	2	2/14 (14.3%)	2/2 (100%)	0/2 (0%)	0/2 (0%)	1/2 (50%)	2/2 (100%)	2/2 (100%)	1/2 (50%)	1/2 (50%)	1/2 (50%)
Type 3 (R type)	4	4/14 (28.6%)	3/4 (75%)	1/4 (25%)	0/4 (0%)	2/4 (50%)	4/4 (100%)	4/4 (100%)	4/4 (100%)	2/4 (50%)	4/4 (100%)
Total	14	14/14 (100%)	11/14 (78.6%)	7/14 (50%)	5/14 (35.7%)	9/14 (64.3%)	13/14 (92.9%)	14/14 (100%)	12/14 (85.7%)	9/14 (64.3%)	13/14 (92.9%)

**Table 7:** Interactions of 5-FU with metal ion in protein.

Type	PDB ID	Metal Ion	Interaction
1	6KLK	Zn	ZNα-F5 (3.6 Å)
1	6L0B	Zn	Znα-O2 (3.0 Å), Znβ-O2 (2.4 Å)
1	8GVZ	Zn	Znα-O2 (3.2 Å), Znβ-O2 (2.7 Å)
2	3NAI	Mg	Mg-N3 (3.0 Å)
3	8IS4	Zn	Znα-N3 (3.0 Å)
